# Cement dust exposure and acute lung function: A cross shift study

**DOI:** 10.1186/1471-2466-10-19

**Published:** 2010-04-14

**Authors:** Zeyede K Zeleke, Bente E Moen, Magne Bråtveit

**Affiliations:** 1 Centre for International Health, University of Bergen, Overlege Danielsens Hus, Årstadveien 21, N-5020 Bergen, Norway; 2 Department of Public Health and Primary Health Care, Occupational and Environmental Medicine, University of Bergen, Kalfarveien 31, N-5018 Bergen, Norway

## Abstract

**Background:**

Few studies have been carried out on acute effects of cement dust exposure. This study is conducted to investigate the associations between current "total" dust exposure and acute respiratory symptoms and respiratory function among cement factory workers.

**Methods:**

A combined cross-sectional and cross-shift study was conducted in Dire Dawa cement factory in Ethiopia. 40 exposed production workers from the crusher and packing sections and 20 controls from the guards were included. Personal "total" dust was measured in the workers' breathing zone and peak expiratory flow (PEF) was measured for all selected workers before and after the shift. When the day shift ended, the acute respiratory symptoms experienced were scored and recorded on a five-point Likert scale using a modified respiratory symptom score questionnaire.

**Results:**

The highest geometric mean dust exposure was found in the crusher section (38.6 mg/m^3^) followed by the packing section (18.5 mg/m^3^) and the guards (0.4 mg/m^3^). The highest prevalence of respiratory symptoms for the high exposed workers was stuffy nose (85%) followed by shortness of breath (47%) and "sneezing" (45%). PEF decreased significantly across the shift in the high exposed group. Multiple linear regression showed a significant negative association between the percentage cross-shift change in PEF and total dust exposure. The number of years of work in high-exposure sections and current smoking were also associated with cross-shift decrease in PEF.

**Conclusions:**

Total cement dust exposure was related to acute respiratory symptoms and acute ventilatory effects. Implementing measures to control dust and providing adequate personal respiratory protective equipment for the production workers are highly recommended.

## Background

The health risks posed by inhaled dust particles are influenced by the deposition pattern of the particles in the various regions of the respiratory tract and by the biological responses exerted by the deposited dust particles. Cement dust irritates the skin, the mucous membrane of the eyes and the respiratory system. Its deposition in the respiratory tract causes a basic reaction leading to increased pH values that irritates the exposed mucous membranes [1-7]. Several studies [8-12] have suggested associations between cement dust exposure, chronic impairment of lung function and respiratory symptoms. A Malaysian study has shown association of total dust exposure and respiratory symptoms such as cough, phlegm, chest tightness and also with lung function indices [9]. A few studies [4,5] have suggested a relationship between exposure to cement dust and acute, respiratory symptoms and changes in lung function. In a cement factory in Ethiopia there was an association between respirable dust and peak expiratory flow recorded after shift [4]. In an exposure-response study, Mwaiselage et al. [5] found that exposure to respirable dust was significantly correlated with percentage cross-shift decrease in peak expiratory flow (PEF) for 29 workers. Mwaiselage et al. [5] also reported that the concentration of respirable dust by mass was approximately 40% of the "total" dust, suggesting that the mass fraction of larger particles is higher than that of smaller particles in cement dust. To investigate the association between a broader size range of the cement particle exposure and acute respiratory effects, we measured "total" dust in this study. Total dust deposits along the whole respiratory tract and might be associated with respiratory symptoms from the upper and lower airways. The thoracic fraction of the total dust may provoke local responses in the tracheobronchial region of the lung [13].

The demand for cement in East Africa is increasing due to new investments in the region's poor infrastructure, notably roads in Kenya and the rebuilding of war-torn southern Sudan and Burundi, as well as Rwanda and Uganda [14]. Ethiopia is no exception. Ethiopian's domestic demand for cement is mainly covered by Mugher, Dire Dawa and Messebo cement factories. In 2008 the total annual production capacity of these three factories was 1.75 million tonnes [15], and the total number of employees was about 2500.

The sections in the cement production process include crusher, crane, raw mill, kiln, cement mill and packing. Limestone and red soil are dried, ground, proportioned and homogenized before being transferred to rotary kilns to be burned. The resulting clinker is pulverized with gypsum at the cement grinding mill to make Portland cement or mixed with additives to make cement with various properties [1,2]. The final product is transferred from the storage silos to the packing section for bagging and loading. The product is largely calcium silicates, aluminates and alumino-ferrites. The final product often has low concentrations of chromium [1]. Exposure to cement dust may occur at most stages of the manufacturing process [1,3], and higher dust concentrations have been reported in the crusher and packing sections than in other sections. [4,5].

Workers in developing countries are often from lower socioeconomic classes and are frequently hired without appropriate training and deployed at work sites without proper personal protective equipment or ventilation [3]. The level of awareness about occupational hazards among factory workers in Ethiopia is limited. Thus, how working in highly dusty environments affects health and safety is a serious concern [4].

This study investigated the associations between current "total" dust exposure, acute respiratory function and respiratory symptoms among cement factory workers in Ethiopia.

## Methods

### Study design and setting

A cross-sectional study was conducted between June and August 2005 at the oldest cement factory in Ethiopia. This factory was selected because no previous study has been carried out to assess dust exposure and respiratory health effects at this location which was expected to be representative for the numerous cement factories still using older technology worldwide. The factory is located 500 km east of the capital, Addis Ababa. It was established in 1935, and production began in 1945. There has been no major change in production technology since the start of production until this study was conducted. During the study period about 34,000 tonnes of cement was produced annually by 320 workers with one day shift of 8 hours.

### Study groups

Everyone studied was male, as no women are employed in production. The workers were classified into two exposure groups according to the expected exposure to cement dust. The exposed production group comprised all workers from the crusher (*n *= 20) and packing sections (*n *= 22). A control group consisted of all guards (*n *= 20) from the gates of the factory, which is 500 meters away from the production area. In the packing section, one worker did not volunteer to participate in the study and one worker was not on duty, thus leaving 20 workers from the packing section in the final study population.

### Exposure to cement dust

Personal "total" dust was measured in the breathing zone of the workers. Sampling was carried out once for all participating workers from the crusher (*n *= 20) and the packing section (*n *= 20) and for 10 of the controls. Measurements were performed either for two or three different workers per day and it took 3 weeks to conduct all the measurements. No repeated measurements were done. In each section sampling was performed in accordance with the order of names in the alphabetical list from the factory. "Total" dust was collected on pre-weighed cellulose acetate filters with a pore size of 0.8 μm placed in a closed face 37-mm filter cassette (Millipore) connected to an SKC sidekick pump with a flow rate of 2.0 l/min. The sampling time varied from 383 to 457 minutes. Total dust was measured quantitatively by gravimetric analysis at X-lab AS (Bergen, Norway), which has passed the Norwegian intercalibration test for dust sample analysis. The sampling pumps were calibrated before sampling using a rotameter. The Millipore filters were weighed before and after sampling on a microbalance (Mettler AT261), with a detection limit of 0.01 mg/m^3^. We have used the threshold limit value (TLV) from the American Conference of Governmental Industrial Hygienists for total dust of 10 mg/m^3 ^[16] as an occupational exposure limit.

### Assessment of respiratory health effects

#### Interview

The workers were interviewed instead of being asked to complete a questionnaire, as their reading skills were not known. The interviews were performed by the first author and an experienced data collector on the same days the workers carried dust samplers. Prior to the fieldwork, the questionnaire was translated from English to Amharic and back-translated to English by two people. One person translated the questionnaire from English to Amharic, and another person translated back to English and then compared according to standard procedures. The introductory part of the questionnaire included age, years of education, years in other industry, years in different sections of the cement factory, use of respiratory protective gear, past respiratory diseases and smoking habits according to the British Medical Research Council questionnaire [17]. Smoking was recorded as "yes" for current smokers and "no" for nonsmokers and ex-smokers. At the end of the day shift, the acute respiratory symptoms experienced that day (cough, shortness of breath, stuffy nose, wheezing, runny nose and sneezing) were scored and recorded on a five-point Likert scale as never (1), mild (2), moderate (3), severe (4) or very severe (5) using a modified respiratory symptom score questionnaire[18].

#### Ventilatory tests

PEF was measured once for all selected workers at their workplace within 20 minutes before and after the shift on the same days they carried dust samplers. PEF was measured in a standing position by using a portable hand-held Mini-Wright PEF meter. PEF was measured on all days of the week and no correction for carryover effect was done. The same investigators who did the interviews measured the PEF. The highest value of three successive technically correct blows was recorded as the final result. Because measuring PEF depends on effort and technique, workers were trained in measurement technique to obtain a valid measure. The percentage acute cross-shift change in PEF was calculated as [(postshift PEF - preshift PEF)/preshift PEF] times 100.

PEF monitoring has been found to be reliable and inexpensive tools in assessing acute lung function in the cement industry with less cost than ordinary spirometer [5].

### Checklist

A walk-through survey was conducted twice daily to check the flow rate of the dust sampler pumps and to observe whether exposed workers in the crusher and packing sections used a respiratory mask or not. The checkup times were from 0700 to 1000 and 1300 to 1500 during the work shift.

### Ethics

The study was approved by the Regional Committee for Medical and Health Research Ethics, Western Norway and the Haramaya University Ethics Committee in Ethiopia. The study design was explained to the management of the factory and to the workers participating in the study. Written consent was obtained from all participating workers.

### Data analysis

The Statistical Package for the Social Sciences (SPSS) version 12.0 for Windows was used for statistical analysis. *P *< 0.05 was used as the criterion for statistical significance. The exposure data were log normally distributed and were log transformed when comparing the levels between the groups and when analysing the relationship between exposure and PEF. Independent and dependent *t*-tests were used to compare continuous variables. Analysis of variance (ANOVA) was used for comparing groups. When this test produced significant results, post-hoc comparisons using the Bonferroni test were used to explore differences between each of the groups. The five-point acute respiratory symptom score was dichotomized into "no" for those who scored never and "yes" for those who scored mild, moderate, severe or very severe as very few reported severe and none reported very severe. Education was dichotomized into primary for those with grade 4 or less and post-primary for those with grade 5 or more. Contingency tables were analyzed using Fisher's exact test. The relationship between exposure and PEF was analysed using multiple linear regression. Logistic regression was not done on symptoms because of low numbers in the low exposed groups.

## Results

### Cement dust exposure

The highest geometric mean dust exposure was found in the crusher section (38.6 mg/m^3^) followed by the packing section (18.5 mg/m^3^) and the guards (0.4 mg/m^3^). The range of exposure was high in both the crusher and packing sections (Table 1). Within each of these sections exposure was highest during cleaning tasks (Table 1). The log-transformed dust levels differed between the crusher, packing and guards (*P *< 0.0005). Post-hoc comparisons indicated no significant difference in exposure level between the crusher and the packing sections, and they were merged and defined as the high-exposed group.

**Table 1:** Personal "total" dust exposure (*n *= 50) among cement factory workers

		Cement dust exposure			
		
	*n*	AM^a ^(SD)^b ^(mg/m^3^)	Median (range) (mg/m^3^)	GM^c ^(GSD)^d ^(mg/m^3^)	% >TLV^e^
**High-exposed groups**					
Crusher	20	48.8 (31.9)	43.2 (8.8-127.8)	38.6 (2.13)	95.0
Cleaning	4	99.1(20.2)	93.4(81.7-127.8)	97.6(1.2)	
Other tasks	16	36.3(19)	40.1(8.8-71.7)	30.6(1.9)	
Packing	20	44.0 (81.2)	15.7 (2.3-371.8)	18.5 (3.59)	60.0
Cleaning	3	175.2(170.2)	80.4(73.4-371.8)	129.9(2.5)	
Other tasks	17	20.7(20.2)	12.6(2.3-62.3)	13(2.7)	
Crusher and packing	40	46.4 (70.0)	35.9 (2.3-371.8)	26.7 (3.01)	77.5
					
**Low-exposed group**					
					
Guards	10	0.5 (0.3)	0.4 (0.18-0.9)	0.4 (1.73)	0

^a^Arithmetic mean. ^b^Standard deviation. ^c^Geometric mean. ^d^Geometric standard deviation. ^e^Percentage of samples exceeding the Threshold Limit Value.

In the crusher and packing sections, 95% and 60% of the "total" dust samples exceeded the TLV, respectively. None of the samples from the control groups exceeded this level.

### Checklist

None of the exposed workers was observed using a proper personal respiratory mask; 15% of the workers in the packing section had some kind of cloth to cover their mouth and nose during their 8-hour work shift, whereas the others did not use any respiratory protective devices.

### Interview

All 60 workers completed the interview and the ventilatory tests. The mean age was 32.2 years (range 20-56). The low exposed workers (guards) were significantly older than the high-exposed workers (crusher and packing) (Table 2). However, the two groups did not differ in education, employment duration, height or current smoking (Table 2). The range of employment years in the cement factory was 1-17 for the high exposed workers and 2-22 for the low exposed workers. Eighty-two percent of the high exposed workers and 70% of the low exposed workers had post-primary education. Both groups had 10% current smokers and 11% ex-smokers. Neither the high exposed nor low exposed groups did report any past respiratory illnesses (Table 2).

**Table 2:** Demographic data on 60 male cement factory workers categorized into groups with high and low dust exposure

Variables	High exposed	Low exposed	P
	(n = 40)	(n = 20)	
Age (years)^a^	28.5 (8.2)	39.7 (7.9)	< 0.0005^c^
Height(m)^a^	1.72 (0.075)	1.72 (0.048)	0.988^c^
Employment (years)^a^	6.8 (5)	9 (7)	0.124^c^
Primary education only^b^	7 (17.5)	6 (30)	0.326^d^
Current smokers^b^	4 (10)	2 (10)	1.000^d^
Non smokers^b^	36(90)	18(90)	1.000^d^
Ex-smokers^b^	4(11)	2(11)	1.000^d^
Past respiratory diseases	0(0)	0(0)	1.000^d^

^a^Mean (standard deviation). ^b^Number (%). ^c^Independent *t*-test. ^d^Fisher's exact test.

Except for cough, the high exposed workers had significantly higher prevalence for all the acute respiratory symptoms than the low exposed workers (Table 3). The result did not change when the analysis was done after excluding smokers. The highest prevalence of respiratory symptoms for the high exposed workers was stuffy nose (85%) followed by shortness of breath (47%) and sneezing (45%). Very few workers in the low exposed group reported acute respiratory symptoms (Table 3).

**Table 3:** Acute respiratory symptoms and peak expiratory flow (PEF) among 60 male cement factory workers

*Acute respiratory Symptoms, n (%)*	Departments		Exposed groups		Significance levels (p)	
	Crusher (n = 20)	Packing (n = 20)	High exposed (n = 40)	Low exposed (n = 20)	High exposed^a ^vs Low exposed	Crusher vs Pacing
Cough	4(20)	8(40)	12(30)	2(10)	0.112^b^	0.168^c^
Wheezing	6(30)	8(40)	14(35)	0(0)	0.002^b^	0.507^c^
Shortness of breath	9(45)	10(50)	19(47)	1(5)	0.001^b^	0.752^c^
Stuffy nose	19(95)	15(75)	34(85)	0(0)	< 0.0005^b^	0.077^c^
Sneezing	9(45)	9(45)	18(45)	1(5)	0.002^b^	1.000^c^
Runny nose	4(20)	6(30)	10(25)	0(0)	0.023^b^	0.465^c^
						
*Peak expiratory*						
*Flow, AM (SD)*				(n = 10)		
Preshift PEF (l/m)	455(84.9)	505(65.2)	480(78.9)	429(83.2)	0.024^d^	0.043^d^
Postshift PEF (l/m)	437(94.3)	483(67.6)	459(84.2)	455(84)	0.85^d^	0.087^d^
ΔPEF%	-4.2(8.9)	-4(10.5)	-4.1(9.6)	6.8(10.7)	< 0.0005^d^	0.949^d^

^a^High exposed = Crusher + Packing, ^b^Fisher's exact tests, ^c^Chi-square tests, ^d^Independent *t-test*

### Ventilatory test

The high exposed group had significantly higher preshift PEF value than the low exposed group (Table 3). Cross-shift change in PEF declined in the high exposed groups (*P *= 0.003), whereas cross-shift change in PEF increased among the low exposed workers (*P *= 0.004). Although the postshift PEF did not differ between the groups, the percentage change in PEF (ΔPEF%) across the shift differed significantly between the groups (Table 3).

**Figure 1:**
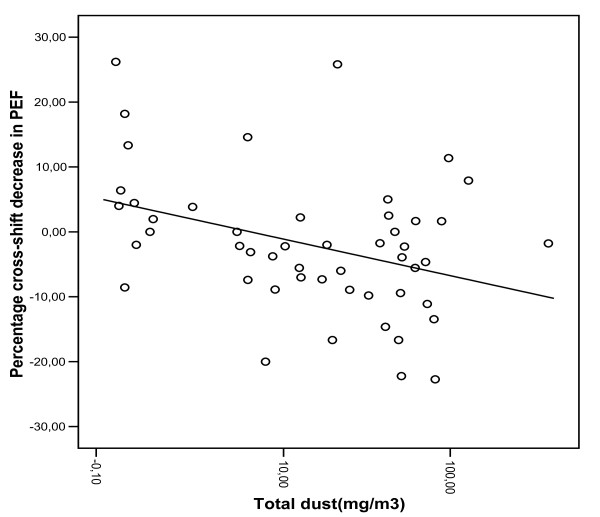
**Scatter plot for the relationship between "total" dust exposure and the percentage cross-shift decrease in PEF for cement factory workers (*n *= 50)**.

The percentage cross-shift change in PEF and total dust exposure were significantly negatively correlated (Table 4, Fig. 1). A regression model including the number of years in high exposed sections (packing and crushing), current smoking and log-"total" dust exposure explained 25.4% (adjusted *R*2) of the variance in the percentage cross-shift change in PEF.

**Table 4:** Multiple linear regression model for percentage cross-shift change in PEF (ΔPEF%) among 50 male cement workers

	Covariates	B	SE	*p*	95%CI
ΔPEF%	Constant	5.46	2.19	0.016	1.05, 9.87
	Years in high-exposed section	-0.80	0.37	0.036	-1.55, -0.05
	Current smoking (0/1)	-6.60	4.45	0.144	-15.53, 2.33
	Log-"total" dust (mg/m^3^)	-1.62	0.73	0.032	-3.09, -0.15

ΔPEF% = [(postshift PEF - preshift PEF)/preshift PEF] times 100. B: regression coefficient. SE: standard error of regression coefficient. 95% CI: 95% confidence interval.

Among the high exposed workers the average number of employment years in the crusher and packing sections, excluding years in other sections, was 4.4 years. Further, each year of work in the packing or crusher section was associated with a decrease in cross-shift PEF of 0.80%. 35% of the guards had been working in the production section prior to being a guard. However, they had been working only for a year or less and we found no significant difference in cross-shift changes between guards who had previously worked in the production and guards who had not.

The results did not change significantly when employment years in the quarry and cement mill sections were added to the number of years in high exposed sections. Height and age were not significantly associated with cross-shift PEF in the multiple regression and were not included in the final model. Age was not correlated with the number of years in high exposed sections, but age and preshift PEF were negatively correlated.

## Discussion

The "total" dust concentrations for the workers in both the crusher and packing sections were significantly higher than for the guards. Moreover, acute respiratory symptoms and the percentage cross-shift decrease in PEF were significantly more pronounced among high exposed workers than among low exposed controls.

The "total" dust exposure in the crusher section was higher than among cement workers in a study from the United Republic of Tanzania (geometric mean (GM): 13.5 mg/m^3^) [18]. Further, the level in the packing section was higher than that in a Malaysian cement factory (GM: 2.1 mg/m^3^) [9] but similar to the level in Tanzania (GM: 21.3 mg/m^3^) [19]. In our study, 95% and 60% of the "total" dust samples in the crusher and packing sections (respectively) exceeded the TLV. This is very high compared with cement workers in the United States [12] in which only 19% of 211 personal total dust samples from the crusher and packing sections exceeded the TLV of 10 mg/m^3^. This may be due to better dust control measures at these workplaces in the United States. Only 15% of the packing section workers in our study had some kind of cloth to cover their mouth and nose, and the cloths were probably not effective in reducing dust exposure. This is similar to the conditions in a cement plant in Nigeria, where the workers did not use any protective devices such as respirators, goggles or gloves [20]. This increases the risk of negative health effects for workers in high exposed areas. The large ranges of exposure in both the crusher and packing sections may be due to different activities or tasks among the workers on the sampling days. Workers performing cleaning activities had higher dust levels compared to other workers in the production sections (Table 1), but they were too few to constitute a separate group for analyzing respiratory effects. According to the workers and their supervisors, the work tasks in the production section on the days of dust sampling were representative for normal working days. None of the samples from the controls exceeded the TLV, probably because the guards were 500 metres from the production area and worked in the open air. The most common selection bias in occupational epidemiology is the healthy worker effect, which refers to overrepresentation of healthy workers in the exposed jobs while ill workers quit. This will inevitably lead to an underestimation of work related disease in the work force. In Ethiopia, as a result of high unemployment rate, workers are probably more likely to continue work even when having reduced health. Although we can not exclude healthy worker effect also in Ethiopia, it is presumably of less importance than observed in high-income countries.

The exposed group had significantly more acute respiratory symptoms than the controls. These effects are presumably associated with the high concentration of dust in the working environment and may be related to the basic reactions caused by the cement dust, which irritate the respiratory tract. Though we didn't adjust for age, the relatively young workers in the production had a higher prevalence of acute respiratory symptoms compared to the older, low exposed guards. The prevalence of respiratory symptoms in some cases is assumed to increase with age [21], thus supporting our suggestion that there is a strong association between cement dust exposure and acute respiratory symptoms. The results of the acute respiratory symptom scores are in agreement with Mwaiselage et al. [5], who found a high prevalence of shortness of breath, stuffy nose and sneezing among exposed cement factory workers.

In our study, a cross-shift decrease in PEF was found only among the high exposed workers. The reduced postshift PEF values in the crusher and packing sections were presumably due to the high concentration of dust in the working environment, in agreement with previous cement studies [4,5]. The higher preshift PEF among the high exposed groups might be due to their young age compared with the control groups, as indicated by the correlation between preshift PEF and age. Further, healthy workers may be selected into the high-exposure jobs. The increase in PEF for the guards across the shift might be due to normal diurnal changes. For none-exposed populations PEF is normally reported to be lowest in the morning and highest in the mid-afternoon [22,23]. This circadian influence on cross-shift variation in PEF has also been noted in control groups whereas exposed stainless-steel workers had a decrease in PEF across the day shift [24].

In the multiple linear regression model, the number of years of employment in the crusher and packing sections were associated with an increased percentage of cross-shift decrease in PEF. This may be caused by increased sensitivity of the airways related to long-term cement dust exposure in general or by hypersensitivity to specific components such as the trace amounts of chromium present in the cement dust, and might be a sign of chronic negative health effects. This should be evaluated further in future longitudinal studies. The prevalence of smoking among the workers was generally small and the same for both high exposed and low exposed groups. Hence it is unlikely to affect the analysis. The observed negative effect of current smoking (-6.6%), although not significant, suggests that smokers react more to the acute dust exposure than nonsmokers (Table 4). Pack years was not reported as the number of smokers were very few.

Mwaiselage et al. [5] found a 14% percentage cross-shift decrease in PEF for a nonsmoker, working for about 11 years and exposed to 10.6 mg/m^3 ^of respirable dust. When assuming that the concentration of respirable dust by mass in our study was also approximately 40% of the "total" dust, our regression equation predicts that the percentage cross-shift decrease in PEF would be 9% for a nonsmoker exposed to 26.5 mg/m^3 ^"total" dust and with 11 years of work experience in high-exposure sections (Data not shown).

Although the findings in this factory cannot be generalized to modern cement factories, they might be representative of the situation in numerous cement factories using older technology in Ethiopia and other countries.

## Conclusions

Workers in the crusher and packing sections were highly exposed to total cement dust relative to TLV, and total dust was related to acute respiratory symptoms and acute ventilatory effects. The acute respiratory health effects can presumably be reduced by proper dust control measures such as personal protective devices (respirators), training and education and maintaining machines at the workplace. Stringent follow-up and providing high-quality personal respiratory protective equipment for the production workers is highly recommended. Further studies to investigate the possible sensitizing effects of cement are needed.

## Competing interests

The authors declare that they have no competing interests.

## Authors' contributions

ZKZ designed and conducted the study, undertook the analysis, made revisions to the manuscript after consultation with the other authors. BEM and MB participated on the design and analysis, conducted review and provided scientific support throughout the project and comments on the manuscript. All authors have read and approved the final manuscript.

## Pre-publication history

The pre-publication history for this paper can be accessed here:

http://www.biomedcentral.com/1471-2466/10/19/prepub
